# Lanolin-Based Synthetic Membranes as Percutaneous Absorption Models for Transdermal Drug Delivery

**DOI:** 10.3390/pharmaceutics10030073

**Published:** 2018-06-21

**Authors:** Victor Carrer, Beatriz Guzmán, Meritxell Martí, Cristina Alonso, Luisa Coderch

**Affiliations:** Department of Chemical and Surfactants Technology, Institute of Advanced Chemistry of Catalonia (IQAC-CSIC), 08304 Barcelona, Spain; bgmtqt@cid.csic.es (B.G.); mmgesl@cid.csic.es (M.M.); cristina.alonso@iqac.csic.es (C.A.); lcnesl@cid.csic.es (L.C.)

**Keywords:** synthetic membranes, lanolin, skin absorption, Franz cell, topical exposure

## Abstract

Background: The major in vitro permeation studies are currently performed in Franz-type diffusion cells because of their simplicity, cost effectiveness and because the experimental conditions can be easily controlled. Apart from the skin, Franz-type diffusion cells can be used with synthetic membranes. Nevertheless, they do not emulate the nature of the lipidic matrix, which is responsible for the topical barrier function. Objective: This paper offers two new approaches combining different synthetic membranes (Strat-M^®^ and Nucleopore^®^) with lanolin, which provides lipidic components similar to the lipidic matrix. Methods: The molecular structure of lanolin was studied in membranes by attenuated total reflectance infrared spectroscopy (ATR-IR). The water permeability and absorption of lidocaine, diclofenac sodium and betamethasone dipropionate were also studied and compared against free-lanolin membranes and skin. Results: The results showed an increasing barrier function after lanolin application in both membranes, resulting in a decrease in water permeability. Observing the IR spectra, the lateral packaging of the lipid in the synthetic membranes seems to emulate the orthorhombic disposition from the stratum corneum. Moreover, the three substances applied to the lanolin-containing membranes have a similar absorption to that of the skin. Conclusions: In conclusion, combining synthetic membranes with lanolin may be a useful approach to mimic topical actives’ absorption.

## 1. Introduction

The primary barrier to dermal drug delivery is the stratum corneum (SC), which forms the outermost layer of the epidermis [1]. The SC consists of several layers of partially-overlapping corneocytes, which are surrounded by the cell envelope and are imbedded in a lipid matrix. Only a limited number of drugs can cross the SC. Drug permeation across the SC depends on the interaction between the skin, the drug and the components in the formulation vehicle [2,3]. Due to the highly impermeable character of the cornified envelope of corneocytes, the SC lipid matrix provides the actual barrier to diffusion of the substance through the skin [4,5,6].

Percutaneous penetration warrants in vivo experiments in humans. These experiments are often morally undesirable, expensive and time consuming. Additionally, high inter- and intra-individual variability is found in the data [7]. Therefore, alternatives to in vivo studies in humans are sought. The suitability of the different in vitro permeability models using excised skin (human or animal) to mimic the in vivo studies has been widely reviewed [8], but obtaining a sufficient supply of excised human and animal skin is often a challenge and tends to be costly.

There have also been attempts to create synthetic membranes that may be used as human skin models to investigate the transdermal diffusion properties of pharmaceutical and cosmetic compounds and formulations [9]. The FDA has encouraged the use of porous synthetic membranes to evaluate the performance of topical products because they act as a support without posing a rate-limiting barrier [10]. Unlike skin, these membranes are inert and do not introduce biological variations. Moreover, the variability subjected to the anatomical site, age, race of the skin donor and skin-biopsy preparation and storage can be overtaken. Such synthetic membranes are composed of a thin sheet of polymeric macromolecules that can control the passage of components through them. They may be composed of synthetic polymers (e.g., polysulfone, polycarbonate) or semi-synthetic cellulose polymers (e.g., cellulose acetate, cellulose nitrate). Despite these synthetic models, more efforts should be carried out to emulate the complex composition of the SC lipidic structure [11].

The lipid lamellae are oriented approximately parallel to the surface of the corneocytes, and they are mainly composed of cholesterol, ceramides and free fatty acids [12,13]. The skin contains at least six ceramide families, which are believed to play a different role in skin properties [14]. Moreover, the free fatty acids cause the skin barrier to have a pH of 5.5, which affects the ionization state of the topically-applied substances. Skin barrier disruption due to topical treatment with surfactants or organic solvents is attributed to a selective/integral depletion or lipidic alteration [15]. The first efforts to study lipidic synthetic membranes and compare to skin absorption were carried out by Landmann in 1984 [16]. More recently, Anwar and colleagues [17] developed a simplified model that should serve to a bridge the gap between the more realistic, but complex model systems and the simple models. The challenge here is to develop models that lend themselves to both molecular-level experiments and simulations.

The structure of lanolin, which mimics the lipidic matrix of the SC by having a similar chemical composition and properties, may offer a suitable strategy to achieve accurate modeling of the skin barrier properties by combination with synthetic membranes. It has been demonstrated that wool wax shares important features with SC lipids: wool wax contains cholesterol, an essential constituent of SC lipids. Cholesterol and its derivatives, ceramides or some free fatty acids have been found in lanolin [18,19,20]. Moreover, wool wax and SC lipids can coexist as solids and liquids at physiological temperatures [21,22,23]. In this study, lanolin was obtained with a closed-loop process to scouring wool fibers designed in a pilot plant called the wool dry scouring process (WDS) [24]. It is important to note that with the WDS process, the resulting lanolin has more polar lipids and hence a more human resemblance [25].

The present study focuses on whether the addition of lanolin to synthetic membranes (Nucleopore^®^ and Strat-M^®^) improves the membrane barrier and emulates porcine skin. Dermatomed pig skin is used in these investigations because pig skin morphology and permeability are similar to those of humans [26], and therefore, it is an accepted model for the skin penetration assessment of cosmetic ingredients [2]. Nucleopore^®^ is a 10-µm-thick filter in which a 0.05-nm pore size is created in a polyethylene terephthalate membrane 25 mm in diameter [27], whereas Strat-M^®^ is an ultrafiltration membrane composed of polyethylene sulfone that is predictive of diffusion in human skin [28].

The water permeability is an indicator of the membrane integrity/barrier function and will be evaluated by measuring the transepidermal water loss (TEWL). Additionally, the lipid structure of the lanolin models is evaluated by attenuated total reflection Fourier transform infrared (ATR-IR) spectroscopy to be compared against the porcine SC lipidic spectra. A highly lipidic order (orthorhombic and hexagonal) has been found in isolated SC [29] and in model mixtures of SC lipids [30,31]. This is strongly related to the SC lipid barrier function considering that numerous studies have shown that the permeability of water and small molecules in healthy skin differs considerably depending on the lipidic order [31,32].

The absorption of three substances widely employed in dermatology with different molecular weights and hydrophilic/hydrophobic balances such as lidocaine, diclofenac sodium and betamethasone dipropionate were compared. Considering that the lipophilicity of the substance is a crucial parameter on its skin absorption, the octanol/water distribution coefficient (LogD) of the three actives is also determined at pH 7.4 (physiological pH) and pH 5.5 (skin surface pH) and is discussed together with the permeation results.

Next, the main aim of this work was to obtain lanolin-based synthetic membranes to be used in skin permeation studies as models of mammalian skin. Lipid structural IR evaluation and water permeation and penetration assays of three topical actives will be performed to determine the effect of lanolin on the membrane skin models.

## 2. Methodology

### 2.1. Materials

The Institutional Review Board and Animal Ethics Committee of the University of Barcelona, Barcelona, Spain, approved the protocol (28 January 2013). Animal handling was approved by our Institutional Review Board and Ethics Committee (approval reference number: DMAH 5605). The management of the animals used in this study conforms to the Guide for the Care and Use of Laboratory Animals published by the United States National Institutes of Health [33] Unboiled porcine skin was obtained from the dorsal area of weanling female white/landrace pigs weighing 30–40 kg. Following euthanasia, the bristles were removed carefully with an animal clipper and were subsequently washed with water. The hair-clipped skin was dermatomed using a Dermatome GA630 system (Aesculap, Tuttlingen, Germany) to a thickness of 500 ± 50 µm, cut into pieces (2.5-cm inner diameter) and then stored at −20 °C until their use.

Two synthetic membranes were studied, as well as the matrix from which the lanolin-containing models originated. StratM^®^ (Merck Millipore, Darmstadt, Germany) and Nucleopore^®^ (Sigma Aldrich, St. Louis, MO, USA) synthetic membranes were purchased. Both membranes were cut into discs that overlapped with the edges of the Franz cell compartment to prevent leakage. 

The lanolins that were applied to the synthetic membranes were extracted from Spanish merino sheep using the WDS process from the European Union’s funding program LIFE 11 (project ENV/ES/588 [24]). Briefly, the wool was scoured with hexane to remove dust impurities, then the hexane solution was centrifuged, and the lanolin was recovered from the hexane with distillation at 35 °C. Finally, lanolin was desiccated to remove the residual water.

To study the potential of the membranes to mimic the skin absorption, they were tested with three actives: lidocaine (Sigma Aldrich, St. Louis, MO, USA), diclofenac sodium (Sigma Aldrich, St. Louis, MO, USA) and betamethasone 17,21-dipropionate (Sigma Aldrich, St. Louis, MO, USA).

### 2.2. Membrane Formation

#### 2.2.1. Strat-M-Lanolin

The addition of lanolin to the Strat-M^®^ membranes was carried out following the next protocol: 100 µL of lanolin (see Section 2.1) 5% in a hexane (Merck, Darmstadt, Germany), ethanol 96% (Merck, Darmstadt, Germany) 2:1 solution were applied three times on the top of the Strat-M^®^ membranes under nitrogen flow (Carburos metalicos, Aranjuez, Spain). Next, these Strat-M-lanolin membranes were stored at 85 °C for 10 min to fix the lipids and dry the membrane.

#### 2.2.2. Nucleopore-Lanolin

Lanolin was added to the Nucleopore^®^ membranes following the more complex procedure for lipid fixation previously detailed by Pullmannová et al. [34], wherein the membranes were hydrated in hexane:ethanol 96% (2:1) and then dried at room temperature. Under nitrogen, 100 µL of lanolin 5% in hexane:ethanol 96% (2:1) were applied on the top. The membranes are stored at 2–6 °C in a vacuum desiccator for 24 h. Finally, the membranes were placed at 85 °C for 10 min, and after 3 h at room temperature, the membranes were ready to use.

### 2.3. Lipidic Conformation Analysis by ATR-IR

The lipidic conformation for pure lanolin, porcine skin, Nucleopore-lanolin and Strat-M-lanolin was studied using ATR-IR spectroscopy. The infrared spectra were obtained using the 360-FTIR spectrophotometer Nicolet Avatar (Nicolet Instruments, Inc., Madison, WI, USA) equipped with an attenuated total reflection (ATR) accessory that used a diamond with an angle of incidence of 45° in a horizontal orientation.

Before analysis, the samples were placed with the SC/lanolin side facing the ATR diamond. To ensure reproducible contact between the sample and crystal, a pressure of 10 kPa was applied to the samples. All analyzed spectra represented an average of 32 scans obtained with a resolution of 4 cm^−1^, and the wavenumber range used was 4000–700 cm^−1^. The peak positions were determined with the aid of OMNIC software Version 7.3 (Nicolet, Madison, WI, USA) using a Gaussian–Lorentzian peak fitting and baselined spectra. Two different peaks were studied: 2850 and 2920 cm^−1^, which are assigned to the CH_2_ symmetric and asymmetric stretching vibration, respectively. Analysis of every sample was made in triplicate.

### 2.4. Water Permeability by TEWL

The commercial synthetic membranes (Strat-M^®^ and Nucleopore^®^), lanolin-containing membranes (Strat-M-lanolin and Nucleopore-lanolin) and excised pig skin were mounted in a static Franz diffusion cell with the SC/lanolin side facing the donor compartment.

The nominal surface area was 1.86 cm^2^, and the receptor chamber capacity was approximately 3 mL. The receptor chamber was filled with receptor fluid. The receptor fluid (RF) was Dulbecco’s phosphate-buffered saline at pH 7.4 (Sigma Aldrich, St. Louis, MO, USA) in Milli-Q water with the addition of 0.04% (*w*/*v*) gentamicin sulfate salt (Sigma Aldrich, St. Louis, MO, USA) and 5% (*w*/*v*) bovine serum albumin (Sigma Aldrich, St. Louis, MO, USA). Air bubbles were carefully removed between the skin and RF with a syringe. Finally, the assembled Franz-type cells were placed in a temperature-regulated water bath on top of a water-resistant magnetic stirring plate and stirred at 400 rpm to maintain receptor fluid homogeneity. The water bath was maintained at 37 °C to obtain a membrane surface temperature of 32 ± 1 °C.

Once the skin and membranes were stabilized in the water bath, the integrity and permeability of all the studied membranes were studied with the transepidermal water loss value (TEWL). This was measured for 2 min using a Tewameter TM300 system (Courage & Khazaka, Cologne, Germany) in every replicate before the beginning of the penetration assay. Nine replicates for every model were evaluated. Once the results were obtained, the Kruskal–Wallis test was applied to detect significant differences between the different models. The Statgraphics plus 5 software (Statgraphics.Net, Madrid, Spain) was used for statistical analyses. Significant differences in the mean values were evaluated by the F test. A *p*-value below 0.1 was considered significant.

### 2.5. Penetration Assay on Static Diffusion Franz-Cell Assembly

Lidocaine, diclofenac sodium and betamethasone dipropionate were selected considering that they are commonly employed in topical formulations. Their respective distribution coefficient (LogD) at pH 7.4 and 5.5, as well as their molecular weight were calculated in silico using the Pipeline Pilot software (Accelrys, San Diego, CA, USA).

To avoid the permeability differences caused by the formulation, the three actives were formulated in propylene glycol. Propylene glycol (PG) (Sigma Aldrich, St. Louis, MO, USA) was added to lidocaine, diclofenac sodium and betamethasone dipropionate at 2%, 0.5% and 1%, respectively (same concentration of commercial topical formulations), and then solicited for 10 min to assure the complete solubility of each active in PG.

Having reached this point, 20 µL of each PG solution were applied to the surface delimited by the donor compartment. PG formulations were studied in triplicate in every membrane. After 24 h, the receptor fluid of all the membranes were collected, extracted and analyzed by high-performance liquid chromatography (HPLC) (see the following section). The extractor solvents of each active are established in Table 1.

In the case of the skin, the different skin layers were separated and analyzed following the ensuing procedure. The skin surface was washed and wiped with a cotton swab to remove any remaining formulation and was extracted into 10 mL of extractor solvent (W). Next, 8 strip preparations were carried out on the surface horny layers of the stratum corneum with adhesive tape (D-Squame, Cuderm Corporation, Dallas, TX, USA) applied under controlled pressure (80 g/cm^2^) using a metallic bar for 5 s. The 8 strips were extracted into 2 mL of extractor solvent (S). Finally, the epidermis (E) was separated from the dermis (D) after heating the skin at 80 °C for several seconds. Both tissues were extracted with 1 mL of extractor solvent. All the skin extracts remained overnight with the extractor solvent and then were shaken for 30 min and subsequently sonicated for 15 min. Before the HPLC analysis, all the extracts were filtered with a 0.45-nm nylon filter (Cameo, Perrysburg, OH, USA). The employed reagents were: acetonitrile (Sigma Aldrich, St. Louis, MO, USA), methanol (Merck, Darmstadt, Germany), buffer phosphate (pH 7) (Merck, Darmstadt, Germany), phosphoric acid (Merck, Kenilworth, NJ, USA) and trifluoroacetic acid (Alfa Aesar, Karlsruhe, Germany).

The amount of the active in the samples was determined by the HPLC methodology validated according to the International Conference on Harmonisation Q2 (R1) guidelines in terms of linearity, accuracy and precision [35]. The HPLC system consisted of a VWR-Hitachi HPLC apparatus with a CM5430 DAD detector, L-2130 Pump, L-2200 Autosampler and an interface device. The flow rate was 1 mL/min under isocratic conditions; the injection volume was 20 μL; and all the analyses were performed under room temperature. The columns, wavelengths and mobile phases for every active are compiled in Table 1.

The results are presented as normalized amounts (%) of permeated substance with its standard deviation. The permeated amount in the case of skin is considered the sum of the epidermis, dermis and receptor fluid. For the rest of the membranes, the amounts were found to be equivalent to those in the receptor fluid. Permeability differences between porcine skin and lanolin membranes were sought with the Kruskal–Wallis test previously described in Section 2.4.

## 3. Results and Discussion

### 3.1. Evaluation of the Lipid Conformation of Membranes by ATR-FTIR

The lipidic order of lanolin, Strat-M-lanolin, Nucleopore-lanolin and pig skin was studied using ATR-FTIR. This technique is a non-invasive technique with a depth penetration of 1 µm that makes it suitable to investigate lanolin or SC without isolation from other layers. For this study, of particular interest were the bands associated with the alkyl chain of the lipids. Information about the conformational order-disorder of the skin lipids can be extracted by the analysis of the 2920 and 2850 cm^−1^ stretching bands. In the case of symmetric CH_2_ stretching, vibrations of 2849 cm^−1^, 2850 cm^−1^ and 2852 cm^−1^ indicate orthorhombic, hexagonal and liquid crystalline, respectively. An increase in the vibrational frequency generally indicates an increase in the disorder. A similar behavior was observed for the asymmetric CH_2_ stretching at 2920 cm^−1^, although the symmetric stretching is more sensitive to the conformation changes.

In our case, the peak position in all the replicates was determined, and their average and standard deviation were calculated (Table 2). All the analyzed samples have a vibrational frequency of approximately 2849 cm^−1^ or less corresponding in all cases to orthorhombic conformations. Low standard deviations were obtained in all the samples. These values agree with the ones corresponding to the natural lipidic packaging of healthy pig skin. Therefore, the two proposed synthetic models present a highly ordered lipidic structure that could emulate the lipidic package of the stratum corneum.

### 3.2. Water Permeability of Membranes

**Figure 1:**
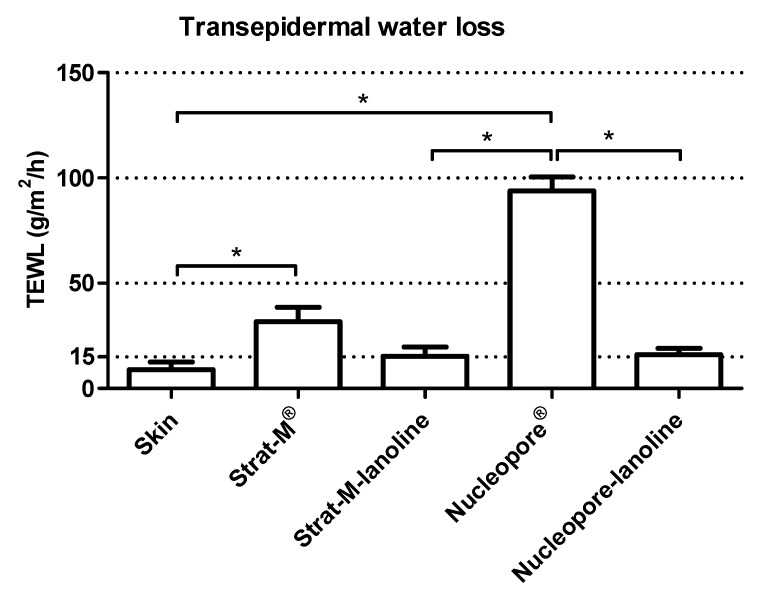
Transepidermal water loss (TEWL) measured on skin, Strat-M^®^, Strat-M-lanolin, Nucleopore^®^ and Nucleopore-lanolin. * indicates a *p*-value ≤ 0.1 from the Kruskal–Wallis test.

Transepidermal water loss (TEWL) is commonly used to measure membrane/skin barrier function permeability and integrity. The loss of water across the membrane is a valuable clue to determine whether a membrane is suitable to test the skin absorption. In this work, the TEWL values and their respective standard deviation have been calculated for each membrane (*n* = 9) before topical application (Figure 1).

A low standard deviation was obtained in all the different membranes, including the skin. This is an important detail considering that it may imply homogeneity and stability not only of the skin biopsy but also of the commercial membranes (Strat-M^®^ and Nucleopore^®^) and the presented lanolin-containing membranes from this work (Strat-M-lanolin and Nucleopore-lanolin).

The skin biopsies were appropriate to test because the values were equal to or below 15 g/m^2^/h [36]. Observing the values obtained from the commercial models, the TEWL values for Strat-M^®^ were lower than those for Nucleopore^®^, indicating a better barrier function for Strat-M^®^. However, their permeability was far away from the maximum accepted for the Organisation for Economic Co-operation and Development (OECD) guidelines. The TEWL results of Strat-M-lanolin and Nucleopore-lanolin indicated that the addition of lanolin brings an important reduction of the TEWL values, making the values comparable to those from the skin. The addition of the lanolin layer on Strat-M^®^ and Nucleopore^®^ sought the empowerment of the barrier function in both membranes, overcoming one of the main problems when mimicking the skin barrier with artificial membranes: the high permeability compared with that of the skin [37]. By adding lanolin to the original synthetic membrane, a significant reduction was observed (*p* ≤ 0.1) when the Kruskal–Wallis test was performed, implying an increase in barrier function.

In summary, the lanolin layer on Strat-M^®^ and Nucleopore^®^ reduced the TEWL values. No significant differences were found between the skin and the two membranes with lanolin after performing the Kruskal–Wallis test (*p* > 0.1). This means there was a similarity between the skin and the lanolin membranes on water permeability.

### 3.3. Physico-Chemical Properties of Actives

Lidocaine, diclofenac sodium and betamethasone dipropionate were selected considering that they are commonly employed in topical formulations and because they have a different chemical nature (basic, acid and neutral, respectively) and a wide range of lipophilicity. Their respective distribution coefficients (LogD) at pH 7.4 and 5.5, as well as their molecular weights were calculated in silico using Pipeline Pilot software (Accelrys, San Diego, CA, USA).

LogD was determined at pH 5.5 because it is the pH of the skin surface, and LogD was determined at pH 7.4 because it is the pH of the receptor fluid and blood. As shown in Table 3, betamethasone dipropionate, as a neutral active, showed no changes in LogD at the different pH values. By contrast, lidocaine (base) and diclofenac sodium (acid) showed opposite behaviors. At lower pH, the increase in the ionized form of the lidocaine caused a decrease in LogD, resulting in higher hydrophilicity. In the opposite case, the LogD at pH 5.5 for diclofenac sodium was higher than that at pH 7.4 because the non-ionized form was predominant at acidic pH. This implied that, at pH 5.5, the LogD range was, from highest to lowest, betamethasone dipropionate > diclofenac sodium > lidocaine, with lidocaine the most hydrophilic compound. However, at pH 7.4, the LogD range was betamethasone dipropionate > lidocaine > diclofenac sodium, with diclofenac sodium the most hydrophilic compound. This could be important to predict or explain the permeation of these actives through the different membranes.

### 3.4. Penetration Assay of Actives through Membranes

As described in the methodology, lidocaine, diclofenac sodium and betamethasone dipropionate solubilized in PG were applied via Franz-cells onto pig skin biopsies, lanolin-free synthetic membranes (Strat-M^®^ and Nucleopore^®^) and lanolin synthetic membranes (Strat-M-lanolin and Nucleopore-lanolin).

When pig skin biopsies were used, the amount of actives was evaluated in the stratum corneum (S), epidermis (E), dermis (D) and receptor fluid (RF). Skin permeation (Perm) was considered the summed amounts from the epidermis, dermis and receptor fluid (Table 4).

**Figure 2:**
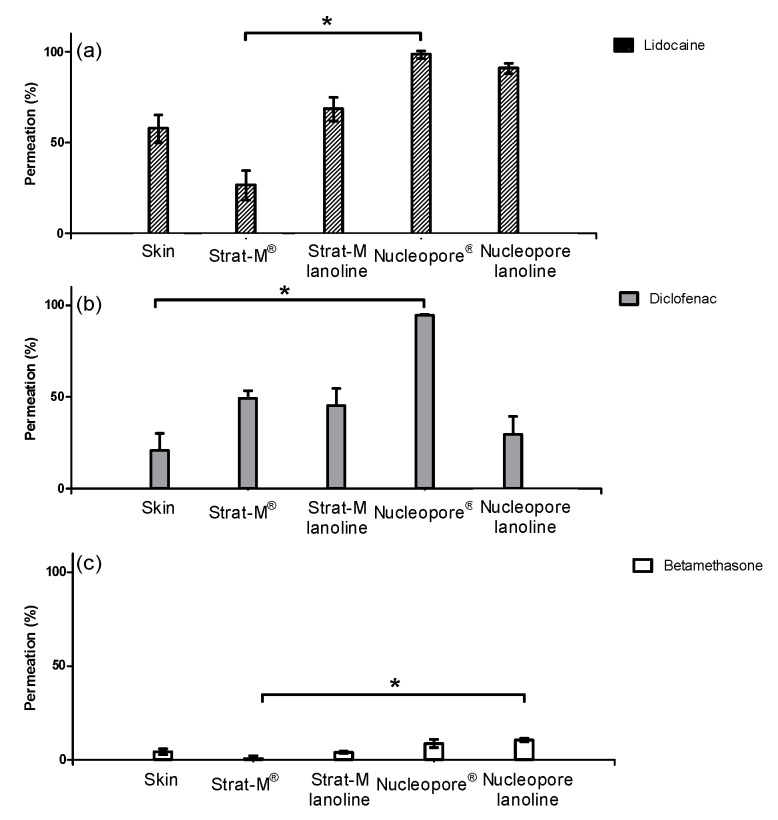
Normalized absorbed amounts (%) in skin, Strat-M^®^, Strat-M-lanolin, Nucleopore^®^, Nucleopore-lanolin for lidocaine (**a**), diclofenac sodium (**b**) and betamethasone dipropionate (**c**). * indicates a *p*-value ≤ 0.1 from the Kruskal–Wallis test.

When the actives were applied on the skin, lidocaine showed the highest permeation rates followed by diclofenac sodium. Betamethasone dipropionate was the less absorbed compound. As explained previously, LogD was an important parameter when studying diffusion across membranes. In the case of skin, LogD at pH 5.5 must be considered because this is the physiological pH in this tissue. When observing the LogD at pH 5.5 and molecular weight for the three actives, the absorption through the skin seemed to be promoted by low molecular weights and low LogD values. Lidocaine (highly absorbed) was small and the most hydrophilic compound, followed by diclofenac sodium with a medium value of LogD and molecular weight. Betamethasone dipropionate was a lipophilic and heavy molecule poorly absorbed across the skin (Figure 2 and Table 4).

The permeation obtained using the commercial membranes confirms the deductions made when observing the TEWL values. We observed higher values of TEWL on Nucleopore^®^ than in Strat-M^®^ that could imply higher permeation on Nucleopore^®^. Hence, the three different actives permeated more into Nucleopore^®^ rather than into Strat-M^®^. These differences were hardly seen on betamethasone dipropionate because the lack of solubility in the acceptor fluid limited its diffusion through the membrane (Figure 2). For the three compounds, the Kruskal–Wallis test could not detect any significant difference (*p* > 0.1) between the skin permeability and the lanolin surrogates.

When observing the actives’ absorption rank on Strat-M^®^, unlike on skin, diclofenac sodium permeated more than lidocaine. Similar results could be deduced for Nucleopore, but its low barrier (demonstrated observing the TEWL values) did not allow the differentiation between lidocaine and diclofenac sodium. These permeability changes between diclofenac sodium and lidocaine could be explained by observing their respective LogD. As discussed previously, the pH of the skin is 5.5, whereas the pH of the commercial membranes was that of the receptor fluid, pH 7.4. Observing their respective LogD, diclofenac sodium was demonstrated to be more hydrophilic than lidocaine at pH 7.4. Therefore, the same deduction observed when the actives were applied on the skin could be extracted: the most hydrophilic compound, which, in this case was diclofenac sodium, permeated more than lidocaine. Betamethasone dipropionate with the highest LogD remained the least permeated compound.

In summary, the lanolin layer was added to Strat-M^®^ and Nucleopore^®^ synthetic membranes. Comparing their TEWL values against those from the lanolin-containing membranes, an increment of the barrier function as a result of the addition of lanolin was observed. The permeation results obtained for the three actives showed this barrier enhancement. The addition of lanolin promoted the reduction in the absorption of the three substances. Moreover, the rank in which the actives were absorbed, lidocaine > diclofenac sodium > betamethasone dipropionate, was identical to that for the lanolin-containing membranes and pig skin one (Figure 2). As stated in the Introduction section, lanolin has a similar composition and pH as the skin lipidic matrix. Therefore, the LogD value that must be considered is 5.5. At this pH, lidocaine was the most hydrophilic active followed by diclofenac sodium and betamethasone dipropionate.

Lanolin addition to the artificial membranes caused a reduction of TEWL and a modulation of the permeation of the three different compounds, leading to results very similar to those obtained with the skin. Lanolin previously demonstrated its ability to reinforce SC lipids, leading to improved skin barrier function in in vivo topical studies [25]. This work confirms its suitability regarding its use as an artificial membrane for permeation or percutaneous absorption models.

## 4. Conclusions

This study demonstrated the potential utility of lanolin addition to artificial membranes Strat-M^®^ and Nucleopore^®^ as a model for the absorption of topical actives. Both membranes were mounted in a vertical diffusion cell, and the transepidermal water loss and percutaneous absorption of three topical actives (lidocaine, diclofenac sodium and betamethasone dipropionate) were compared against the membranes without lanolin and porcine skin. To better understand the permeability changes of the actives in the different models, the active lipophilicities were determined in silico at pH 5.5 and 7.4, corresponding to the skin surface and systemic environment, respectively.

Results have shown that the addition of lanolin to synthetic membranes significantly reduces the TEWL values, similar to the porcine skin levels. The lipid conformation and structure studied by ATR-IR indicate a high lipidic order and orthorhombic structure for lanolin synthetic membranes, similar to the lipidic structure of the skin. The Franz-cell absorption study showed that the hydrophilicities of the actives were closely related to their diffusion through the membranes. Lanolin addition to the artificial membranes caused a reduction of TEWL and modulation of the permeation of the three different compounds, leading to results very similar to those obtained with the skin. Either in the TEWL or the penetration results, no significant differences were observed between the skin and the lanolin-membranes. In conclusion, combining synthetic membranes with lanolin may be a useful approach to mimic the absorption of topical actives. Future steps should include other studies of membrane integrity such as transepithelial resistance and the percutaneous absorption of other formulations and actives.

## Figures and Tables

**Table 1:** HPLC analytical conditions for lidocaine (Lid), diclofenac sodium (Dic) and betamethasone dipropionate (Bet).

Compound	Lidocaine	Diclofenac Sodium	Betamethasone Dipropionate
Extractor solvent	ACN-TFA 0.5%	Methanol	Methanol
Column	LiChrocart^®^ 125-4	LiChrocart^®^ 250-4	LiChrocart^®^ 250-4
	Lichrosphere^®^ 100RP-18	Lichrosphere^®^ 100RP-18	Lichrosphere^®^ 100RP-18
	5 µm	5 µm	5 µm
Wavelength (nm)	205	254	239
Injection volume (µL)	20	20	20
Mobil phase	70% buffer phosphate pH 7.4 30% acetonitrile	66% methanol 34% phosphoric acid 0.7%	73% methanol 27% water
Linear regression equation (R^2^)	A=414,046 [Lid]−68,532 (0.9999)	A=80,050 [Dic]−2484 (0.9997)	A=1,811,416[Bet]+47,781 (0.9999)

**Table 2:** ATR-FTIR of CH_2_ symmetric and asymmetric stretching modes of pure lanolin, Nucleopore-lanolin, Strat-M-lanolin and pig skin.

	Lanolin	Nucleopore-Lanolin	Strat-M-Lanolin	Pig Skin
**λCH2 St. Sym**	2848.5 ± 0.01	2848.5 ± 0.10	2848.5 ± 0.03	2849.4 ± 0.03
**λCH2 St. Asym**	2917.9 ± 0.07	2917.9 ± 0.19	2916.3 ± 0.03	2916.9 ± 0.08

λCH2 St. Sym: CH2 symmetric stretching; λCH2 St. Asym: CH2 asymmetric stretching.

**Table 3:** Molecular weight (MW) and octanol water distribution coefficients (LogD) at pH 5.5 and 7.4 obtained from the ChemAxon platform.

Active (Acidic Nature)	LogD at pH 5.5	LogD at pH 7.4	MW (g/mol)
Lidocaine (basic)	0.61	2.33	234.34
Diclofenac sodium (acid)	2.75	1.1	318.14
Betamethasone 17,21-dipropionate (neutral)	3.96	3.96	504.59

**Table 4:** Normalized amounts (%) found in the stratum corneum (SC), epidermis (E) dermis (D) and receptor fluid (RF). In the case of artificial membranes, skin permeation (Perm) belongs to the amounts found in the receptor fluid.

Normalized Amounts (%)		Lidocaine 2%	Diclofenac Sodium 0.5%	Betamethasone Dipropionate 1%
**Skin**	**S**	5.02 ± 4.56	9.70 ± 4.25	13.60 ± 3.37
	**E**	4.61 ± 3.63	7.34 ± 2.22	4.30 ± 1.58
	**D**	2.94 ± 0.96	1.84 ± 1.53	0.00 ± 0.01
	**RF**	50.02 ± 10.05	11.68 ± 6.17	0.00 ± 0.00
	**Perm**	57.58 ± 7.63	20.86 ± 9.24	4.30 ± 1.57
**Strat-M^®^**	**Perm**	26.48 ± 8.14	49.19 ± 4.23	0.78 ± 1.36
**Strat-M-lanolin**	**Perm**	68.40 ± 6.50	45.22 ± 9.36	4.03 ± 0.59
**Nucleopore^®^**	**Perm**	98.37 ± 2.10	94.56 ± 0.45	8.69 ± 2.16
**Nucleopore-lanolin**	**Perm**	90.81 ± 2.84	29.58 ± 9.78	10.54 ± 0.99
